# Neurobiological Correlates of Alpha-Tocopherol Antiepileptogenic Effects and MicroRNA Expression Modulation in a Rat Model of Kainate-Induced Seizures

**DOI:** 10.1007/s12035-018-0946-7

**Published:** 2018-02-22

**Authors:** Patrizia Ambrogini, Maria Cristina Albertini, Michele Betti, Claudia Galati, Davide Lattanzi, David Savelli, Michael Di Palma, Stefania Saccomanno, Desirée Bartolini, Pierangelo Torquato, Gabriele Ruffolo, Fabiola Olivieri, Francesco Galli, Eleonora Palma, Andrea Minelli, Riccardo Cuppini

**Affiliations:** 1 Department of Biomolecular Sciences, Section of Physiology, University of Urbino Carlo Bo, I-61029 Urbino, Italy; 2 Department of Gastroenterology, Marche Polytechnic University, Ancona, Italy; 3 Department of Molecular and Clinical Sciences, Marche Polytechnic University, Ancona, Italy; 4 Department of Pharmaceutical Sciences, University of Perugia, Perugia, Italy; 5 Department of Physiology and Pharmacology, University of Rome Sapienza, Rome, Italy; 6 Center of Clinical Pathology and Innovative Therapy, INRCA-IRCCS, Ancona, Italy

**Keywords:** Vitamin E, Epilepsy, Neuroprotection, Spontaneous recurrent seizures, MicroRNA

## Abstract

Seizure-triggered maladaptive neural plasticity and neuroinflammation occur during the latent period as a key underlying event in epilepsy chronicization. Previously, we showed that α-tocopherol (α-T) reduces hippocampal neuroglial activation and neurodegeneration in the rat model of kainic acid (KA)-induced status epilepticus (SE). These findings allowed us to postulate an antiepileptogenic potential for α-T in hippocampal excitotoxicity, in line with clinical evidence showing that α-T improves seizure control in drug-resistant patients. To explore neurobiological correlates of the α-T antiepileptogenic role, rats were injected with such vitamin during the latent period starting right after KA-induced SE, and the effects on circuitry excitability, neuroinflammation, neuronal death, and microRNA (miRNA) expression were investigated in the hippocampus. Results show that in α-T-treated epileptic rats, (1) the number of population spikes elicited by pyramidal neurons, as well as the latency to the onset of epileptiform-like network activity recover to control levels; (2) neuronal death is almost prevented; (3) down-regulation of claudin, a blood–brain barrier protein, is fully reversed; (4) neuroinflammation processes are quenched (as indicated by the decrease of TNF-α, IL-1β, GFAP, IBA-1, and increase of IL-6); (5) miR-146a, miR-124, and miR-126 expression is coherently modulated in hippocampus and serum by α-T. These findings support the potential of a timely intervention with α-T in clinical management of SE to reduce epileptogenesis, thus preventing chronic epilepsy development. In addition, we suggest that the analysis of miRNA levels in serum could provide clinicians with a tool to evaluate disease evolution and the efficacy of α-T therapy in SE.

## Introduction

Seizures are common neurological symptoms that may require treatment, but not all seizures evolve into epilepsy. The assertion “seizures beget seizures” involves a role of epileptic activity in initiating the epileptogenic process but also in maintaining it, moving toward a chronicity of epilepsy [1]. Between the initial event and the onset of chronic epilepsy, there is a gap of variable duration, currently referred to as latent period, during which several changes occur in brain structures that are associated with the alteration of network excitability and synchronization and may account for epileptogenesis [1, 2]. Indeed, an initial epileptogenic insult can cause cell loss followed by synaptic reorganization of surviving neuronal elements, axonal sprouting within excitatory pathways amplified by loss of inhibitory interneurons, and anomalous neurogenesis [3]; all these rearrangements may induce the emergence of aberrant excitatory and inhibitory connections, leading to epileptiform hypersynchronization. In addition, gliosis, glial alterations, cytokine production, such as IL-1β and TNF-α, and thus neuroinflammation can alter neuronal excitability and synchronicity, by modulating receptor function and expression, contributing to the chronicity of epilepsy [4–7]. These changes, which may be referred to as maladaptive plasticity [8], have been found in animal models of epilepsy that mimic the typical sequence of initial event–latent period–chronic epilepsy, but also in temporal lobe tissue samples from patients who underwent surgery for drug-resistant mesial temporal lobe epilepsy (MTLE) [9]. In recent years, numerous antiepileptic drugs (AEDs) have been developed, but several unmet clinical needs still remain, including resistance to AEDs found in about 30% of patients, adverse effects elicited by AEDs that can further reduce quality of life, and the lack of treatments that can prevent development of epilepsy in patients at risk, by reducing seizure-triggered maladaptive neural plasticity underlying epileptogenic processes.

Since a wealth of studies have underscored a rise in oxidative stress in epilepsy, thus asserting that free radicals can act as a pathogen in the disease [10, 11], natural compounds with antioxidant properties were considered in preventing seizure-induced pathology [12, 13]. Among these, vitamin E was proved to have beneficial effects in epilepsy, i.e., attenuating convulsive behavior and brain oxidative stress [14, 15]. Indeed, as shown in diverse experimental models, pretreatment with vitamin E reduces seizure-induced oxygen and nitrogen free radicals generation on a time scale of minutes to hours [15–18], thereby decreasing the severity of seizures and their detrimental effects. In addition, patients showing resistance to AEDs have benefited from vitamin E treatment, improving seizure control [19, 20]. In all these studies, the antioxidant role of vitamin E was assumed to be accountable for its effects on epileptic disease. However, as described above, neuroinflammation is also involved in the pathophysiology of epilepsy, contributing to epileptogenesis [21, 22] and vitamin E, mainly α-tocopherol (α-T, the isoform showing the highest in vivo biological activity and bioavailability), has numerous compelling non-antioxidant actions [23], suggesting a possible antioxidant independent mechanism involved in mediating its effects on epilepsy. Furthermore, a role of vitamin E has also been demonstrated in regulating cell signaling through microRNA (miRNA) modulation [24]. MiRNAs are small non-coding RNAs able to regulate gene expression targeting many mRNAs. In this way, miRNAs can also regulate transcription factor expression, modulating in turn all cellular pathways, including inflammatory cascades. Some miRNAs involved in the modulation of inflammatory process, i.e., miR-146a and miR-124, were recently identified as epigenetic modulators of seizure and epilepsy in animal models. The brain delivery of a synthetic mimic of miR-146a, the master modulator of the TLR4/NF-kB pro-inflammatory intracellular signaling, was able to reduce the network excitability in the hippocampus of a mouse model of epileptogenesis [25]. MiR-124 was investigated as a candidate dual regulator of Neuron Restrictive Silencer Factors (NRSF) and inflammatory pathways, showing that it is aberrantly expressed during epileptogenesis [26]. In addition, miR-126, one of the most highly expressed miRs in endothelial cells, plays a crucial role in blood–brain barrier integrity [27], which is impaired during epileptogenesis [28].

Recently, we demonstrated that post-seizure administration of α-T for 4 days [29] quenches the neuroinflammatory and neurodegenerative processes of kainic acid-induced status epilepticus (SE), by reducing neuron degeneration and spine loss and decreasing astrocytosis and microglia activation. These findings could be consistent with the hypothesis that α-T antiepileptogenic potential may be ascribed to additional mechanisms beyond the antioxidant one. Here, we extended the α-T treatment throughout the latent period to further investigate the antiepileptogenic potential of α-T and the mechanisms involved. Thus, we used an acute administration of kainic acid as chemoconvulsant agent, which induces a state of prolonged SE, followed by spontaneous recurrent seizures beginning after a latency of about 15 days [30–32]. Therefore, we decided to investigate the excitability of hippocampus circuitry in adult rat with and without α-T post-ictal treatment at this time point (15 days after SE). To address this issue, we performed electrophysiological field recordings in CA1 of brain slices from kainate-treated rats, to assess population spikes, as an index of susceptibility to develop further spontaneous seizures [33]. Brain slices were then challenged with GABA_A_-receptor antagonist bicuculline and the potassium channel blocker 4-aminopyridine to induce epileptiform activity to evaluate seizure threshold. In addition, influence of α-T treatment on GABA and AMPA currents were also investigated in *Xenopus* oocytes microinjected with hippocampal membranes from adult rat with and without α-T post-ictal treatment.

Neuroinflammation markers, oxidative stress, and neurodegeneration were assessed in hippocampi from the different experimental groups. Finally, we tested the potential relevance of selected circulating miRNAs as epileptogenesis biomarkers.

## Materials and Methods

### Animals

Adult male Sprague-Dawley albino rats (Charles River, Italy) were used in accordance with the Italian law on animal experimentation (D.lgs 26/2014; research project permitted with authorization N. 465/2015-PR by Italian Ministry of Health).

Rats (*n* = 72) were administered an acute intraperitoneal (i.p.) injection of kainic acid (KA, 10 mg/kg b.w. in physiological saline) and seizure behavior was observed and scored according to the Racine scale, up to the manifestation of a full SE [34]. Briefly, typical wet dog shakes, full limbic motor seizures, including rearing and loss of postural control, sequentially appeared, and approximately 3 h after KA injection, animals showed profuse salivation, circling and jumping, and SE. Three hours after the appearance of SE, crises were interrupted by diazepam (2 mg/kg b.w.). Animals showing all the Racine scale steps up to the overt status epilepticus were considered (*n* = 48); about 20% of the kainate-injected rats died during or early after the SE, while approximately 14% did not clearly show the full progression of Racine stages. Epileptic rats were randomly divided in two groups: (1) animals to be treated with an i.p. bolus of α-T once a day up to the 15th day (*n* = 24; αK) (250 mg/kg b.w. for the first 4 days followed by 2 mg/kg for the remaining days), (2) rats (*n* = 24; VK) injected with vehicle. In addition, other animals (*n* = 42) were injected with physiological saline instead of kainic acid (non-epileptic rats): half of them (*n* = 21; αC) were treated with α-T (once a day, for 15 days following the same schedule described above) and the other half (*n* = 21; VC) with vehicle. Epileptic rats were monitored, using the Racine scale steps, for spontaneous seizure arising up to the conclusion of treatment protocols. Rats belonging to all the groups were anesthetized and sacrificed as described below.

### Electrophysiological Analyses

#### Schaffer Collateral-CA1 Field Recordings

Kainate-exposed (epileptic—αK, VK) and saline-injected (non-epileptic—αC, VC) rats (*n* = 8 for each group) were anesthetized with ketamine (65 mg/kg b.w., via i.p.) and killed by decapitation. Brains were quickly removed and parasagittal, 400-μm-thick slices were obtained as previously described [35]. Field potential recordings were carried out following a slice equilibration period in the interface-type recovering chamber maintained at room temperature.

Recording and bipolar stimulating electrodes were prepared and filled with artificial cerebrospinal fluid as previously described [35]: the former electrode was placed in CA1 pyramidal cell layer and the latter in the *stratum radiatum*, at approximately 500 μm of distance between them. Slices giving extracellular field excitatory postsynaptic potentials (fEPSPs) of at least 1 mV in amplitude were considered for recordings. To test basal synaptic transmission, input/output curves were obtained applying to the slice square pulses of current (300 μs in duration) with A385 stimulus isolator (World Precision Instruments, USA): fEPSPs were elicited in response to single electrical stimuli of increasing magnitude (from 0 to 160 pA, increments of 20 pA). Then, baseline responses (60% of maximal fEPSP amplitude) were evoked using low-frequency test pulses (at 30-s intervals) and recorded over 30 min, a period sufficient to ensure stability. Population spikes rising up from fEPSP were analyzed.

After a stable baseline was obtained, GABA_A_-receptor antagonist bicuculline (BMI, 50 μM) and potassium channel blocker 4-aminopyridine (4-AP, 50 μM) were added to bath perfusion for 30 min, during which spontaneous drug-induced extracellular field potential events (interictal) and fEPSPs were recorded. The latency to the onset of epileptiform-like activity was estimated.

Electrophysiological data analyses were performed offline using a WinWCP software (Strathclyde electrophysiology software, John Dempster, University of Strathclyde, UK). Experiments and data analyses were performed in blind by the operators.

#### GABA_A_R and AMPA Current Evaluation in Transfected ***Xenopus*** Oocytes

##### Tissue Collection and Membrane Preparation

Brain tissue from αK and VK rats (*n* = 3 for each group) was snap frozen immediately after collection in liquid nitrogen and stored at − 80 °C until further use. The preparation of rat membranes, their injection in *Xenopus laevis*, GABA and AMPA current recordings in oocytes expressing rat functional receptors was carried out as previously described [36]. The use of female *Xenopus laevis* frogs conformed to institutional policies and guidelines of the Italian Ministry of Health (authorization no. 78/2015-PR).

##### Electrophysiology

Twelve to 48 h after injection, membrane currents were recorded from voltage clamped oocytes by using two microelectrodes filled with 3 M KCl. Oocytes were placed in a recording chamber (volume 0.1 ml) and perfused continuously, 9–10 ml/min, with oocyte Ringer’s solution (OR) at room temperature (20–22 °C).

GABA current rundown was defined as the percentage decrease of the current peak amplitude after six 10-s applications of GABA 500 μM at 40-s intervals [36, 37].

Current–voltage (*I*–*V*) relationships were constructed holding the oocytes at − 60 mV and stepping the membrane potential for 2–4 min at the desired value before applying the neurotransmitter. For these experiments, electrodes were filled with K-Acetate (3 M—[38]) to reduce the leakage of a high concentration of Cl^−^ from electrodes into the oocytes. However, the experiments gave the same results when KCl filling solution was used (not shown). To determine the E_GABA_, *I*–*V* relationships were fitted with a linear regression curve-fitting (Sigmaplot 12) software.

Unless otherwise indicated, for all the experiments, GABA 500 μM was applied for 4 s.

In experiments involving AMPA currents, the oocytes were pretreated for 20 s with cyclothiazide (CTZ, 20 μM), before application of 10 s of AMPA, 20 μM [39]. Experiments involving 1 trimethylammonio-5-(1-adamantane-methyl-ammoniopentane dibromide) (IEM 1460, a voltage-dependent open channel blocker which preferentially blocks GluA2-lacking AMPARs; [40]) were performed holding the oocytes at − 80 mV; the current inhibition was calculated as the ratio of the current blocked by IEM 1460 (I GluA2-lacking) over the total I_AMPA_, expressed as a percent.

Chemicals were dissolved in sterile water (GABA, AMPA, IEM 1460) or DMSO (CTZ) and stocked at − 20 °C until use. For all the experiments, solutions were freshly prepared and drugs and neurotransmitters were diluted to the desired concentration in OR. The composition of OR was the following: 82.5 mM NaCl; 2.5 mM KCl; 2.5 mM CaCl_2_; 1 mM MgCl_2_; and 5 mM HEPES, adjusted to pH 7.4 with NaOH. The final concentration of DMSO was always lower than 1:2000 after dilution. All drugs were purchased from Tocris Bio-science (Minneapolis, MN, USA) and OR salts from SIGMA (Saint Louis, MO, USA).

### Biochemical Analyses

Hippocampi from 16 rats (*n* = 4 for each group), killed by an overdose of sodium tiopenthal via i.p., were quickly excised, after transcardial perfusion with ice-cold physiological saline, and stored at − 80 °C up to use. Hippocampi were then homogenized and lysed with 0.5 ml of ice-cold lysis buffer [50 mM Tris–HCl, pH 7.8, 0.25 M sucrose, 1% (*w*/*v*) SDS, 1 μg/ml pepstatin, 10 μg/ml leupeptin, 2 mM sodium orthovanadate, 10 mM NaF, 5 mM EDTA, 5 mM *n*-ethylmaleimide, 40 μg/ml phenylmethylsulfonyl fluoride, and 0.1% Nonidet-P40] and sonicated for 45 s at 50 W. Oxidative stress evaluation and protein expression quantification were performed.

#### Oxidative Stress Evaluation

##### Spectrophotometric Detection of Protein Carbonyls (PCO)

To determinate the level of oxidative stress, we used the measurement of PCO in hippocampus samples using the molecule 2,4-dinitrophenylhydrazine (DNPH) that is a specific probe able to react with PCO leading to the formation of protein-conjugated dinitrophenylhydrazones (DNP). These protein–DNP adducts are characterized by a peak absorbance at 366 nm, which makes DNPH employment easy and useful to perform a quantitative determination of PCO content by spectrophotometer.

A step-by-step protocol to assay PCO was carried out in accordance with [41]. Spectrophotometric measurements of PCO content were performed at 366 nm, and results were expressed as nanomoles of PCO per milligram of protein (nmol/mg protein).

#### Electrophoresis and Western Blotting Analysis

Hippocampus samples were boiled for 4 min and then centrifuged for 10 min at 14,000×*g* to remove insoluble debris. Supernatants were mixed 1:1 (vol/vol) with sample buffer (0.5 M Tris–HCl, pH 6.8, 2% SDS, 10% glycerol, 4% of 2-mercaptoethanol, and 0.05% bromophenol blue) and 30 μg of sample proteins was loaded onto 10, 12, and 15% SDS–polyacrylamide slab gels and subjected to electrophoresis. Prestained molecular mass markers (Bio-Rad, Milan, Italy) were run on adjacent lanes. The gels were electroblotted and stained with Coomassie brilliant blue R250. For immunoblotting, the following antibodies were used: mouse monoclonal antibodies against Claudin 5 (Thermo Fisher Scientific, Waltham, Massachusetts, USA), GFAP (Sigma, Saint Louis, MO, USA), interleukin-1 β (IL-1β; Santa Cruz Biotechnology, Santa Cruz, CA, USA), and interleukin-6 (IL-6; Santa Cruz Biotechnology, Santa Cruz, CA, USA); rabbit polyclonal anti-tumor necrosis factor-α (TNF-α; Sigma) anti-IBA1 (Wako Life Sciences Inc., USA) and anti-actin (Sigma, Saint Louis, MO, USA). Blots were incubated with specific primary antibodies (1:1000) and subsequently with the appropriate secondary antibodies conjugated with horseradish peroxidase (1:3000; Bio-Rad, Milan, Italy). Immune complexes were visualized using an enhanced chemiluminescence Western blot analysis system (Amersham-Pharmacia, Milan, Italy) following manufacturer’s specifications. Blot images were then digitized (Chemidoc, Bio-Rad) and areas of all labeled bands were quantified using the computerized imaging system software (QuantityOne; Bio-Rad). After antibody probing, nitrocellulose membranes were stripped for 30 min at 50 °C with stripping buffer (62.5 mM Tris–HCl, pH 6.7, containing 10 mM β-mercaptoethanol and 2% SDS) and reprobed with anti-actin (1:200). Immune complexes were visualized using an enhanced chemioluminescence. In each series, relative optical densities (arbitrary units) were normalized for densitometric values obtained from actin-labeled bands.

### MICRORNA Expression Analyses

#### Total RNA Extraction from Serum and Hippocampal Homogenate

Peripheral blood samples were collected from anesthetized rats (sodium tiopenthal, 45 mg/kg b.w. via i.p.; *n* = 3 for each group) in appropriate tubes and, following 30 min resting at RT, serum was obtained by centrifugation at 2500 rpm for 5 min at 4 °C. The synthetic *Caenorhabditis elegans* miR, cel-miR-39, was spiked into rat serum before RNA extraction.

Hippocampi were then excised from the anesthetized rats after killing and microRNAs were isolated from supernatant of homogenates prepared as described above (see biochemical analysis paragraph).

After two subsequent spins, total RNA was extracted from 100 μl of rat serum and homogenates using an RNA purification kit (Norgen Biotek Corporation, Thorold, ON, Canada). RNA was stored at − 80 °C until use.

#### Quantitative Real-Time PCR (qRT-PCR) for Mature MicroRNA Analysis

As previously performed [42, 43], microRNA (miRNA) expression was quantified using a real-time approach with the TaqMan miRNA reverse transcription kit and a miRNA assay (Applied Biosystems, Foster City, CA). The TaqMan MicroRNA reverse transcription kit was used to reverse transcribe the total RNA following manufacturer’s instructions. Briefly, 5 μl of RT mix contained 1 μl of each miR-specific stemloop primer (miR-126, miR-146a, and miR-124), 1.7 μl of input RNA, 0.4 μl of 10 mM dNTPs, 0.3 μl of reverse transcriptase, 0.5 μl of 10× buffer, 0.6 μ of RNAse inhibitor diluted 1:10, and 0.5 μl of H_2_O. The mixture was incubated at 16 °C for 30 min, at 42 °C for 30 min, and at 85 °C for 5 min. Afterwards, qRT-PCR was performed in 20 μl of PCR mix containing 1 μl of 20× TaqMan MicroRNA assay (containing PCR primers and probes; 5′-FAM), 10 μl of 2× TaqMan Universal PCR Master Mix No UNG (Applied Biosystems), 1.33 μl of reverse-transcribed product, and 7.67 μl of nuclease-free water. The reaction was first incubated at 95 °C for 10 min, followed by 40 cycles at 95 °C for 15 s and at 60 °C for 1 min. To obtain accurate and reproducible results, the relative expression of circulating miRNAs was quantified using synthetic *C. elegans* miRNA (cel-miR-39) as the reference miRNA.

MicroRNA expression in homogenate was evaluated using U6 as the reference. Each reaction was performed in duplicate.

The qRT-PCR was performed on an ABI PRISM 7500 Real-Time PCR System (Applied Biosystems). Data were analyzed by a 7500 system software (1.1.4.0) with the automatic comparative threshold (Ct) setting to adapt baseline. Detection thresholds were set at 35 Ct. The relative amount of miR-146a, miR-126, and miR-124 was calculated using the Ct method:

$$ \mathrm{Serum}:\Delta \mathrm{Ct}=\mathrm{Ct}\ \left(\mathrm{miR}-146\mathrm{a}/\mathrm{miR}-126/\mathrm{miR}-124\right)-\mathrm{Ct}\ \left(\mathrm{cel}-\mathrm{miR}-39\right);2{-}^{\Delta \mathrm{Ct}} $$

$$ {\displaystyle \begin{array}{l}\mathrm{Homogenate}:\Delta \Delta \mathrm{Ct}=\left[\mathrm{Ct}\left(\mathrm{miR}-146\mathrm{a}/\mathrm{miR}-126/\mathrm{miR}-124\ \mathrm{treated}\ \mathrm{sample}\right)-\mathrm{Ct}\left(\mathrm{U}6\ \mathrm{treated}\ \mathrm{sample}\right)\right]-\\ {}\left[\mathrm{Ct}\left(\mathrm{miR}-146\mathrm{a}/\mathrm{miR}-126/\mathrm{miR}-124\ \mathrm{control}\ \mathrm{sample}\right)-\mathrm{Ct}\ \left(\mathrm{U}6\ \mathrm{control}\ \mathrm{sample}\right)\right];2{-}^{\Delta \Delta \mathrm{Ct}}\end{array}} $$

Results are expressed in the figures as fold change related to control sample (VC) and values less than 1 indicated down-regulation, whereas values higher than 1 indicated up-regulation.

#### MicroRNA Fluorescence In Situ Hybridization (FISH)

Twelve rats (*n* = 3 for each group) were deeply anesthetized with sodium tiopenthal (via i.p.) and transcardially perfused with physiological saline followed by 4% paraformaldehyde (PFA) in phosphate buffered saline (PBS, 0.1 M; pH 7.4). Brains were removed, postfixed in 4% PFA for 48 h, and then transferred in PBS. Brains were embedded in paraffin and cut by a rotative microtome in 6-μm-thick coronal sections, which were processed for in situ hybridization, using TSA™ Plus Fluorescence systems through the miRCURY LNA™ ISH optimization Kit (Exiqon, Euroclone, Italy); procedure for FISH detection of microRNA was performed according to manufacturers’ instructions. Every step of the procedure, including tissue sectioning, took place in a clean and nuclease-free environment. Briefly, PFA-fixed, paraffin-embedded sections were mounted on slides, dewaxed, rehydrated with PBS, and treated with Proteinase K solution at 37 °C for 20 min at the concentration of 2 μg/ml in a Dako Hybridizer machine. Slides were then dehydrated in ethanol and air-dried. The FISH probes (LNA™ microRNA probe, LNA™ scrambled microRNA probe, and LNA™ U6 snRNA to set optimal hybridization conditions) were denatured at 90 °C for 4 min, before proceeding with the hybridization of the probe with the specimen on the slides; a coverslip was applied, sealed with rubber cement, and sections were incubated at 55 °C for 1 h in a Dako Hybridizer machine. Then, after coverslip removal, the slides were placed in a glass jar containing SSC solutions, put into a water bath set to the hybridization temperature to ensure sufficient stringency. Following PBS washes, the slides were placed in a humidifying chamber and incubated with blocking solution (containing 2% sheep serum, 0.1% Tween-20, 1% BSA in PBS) for 15 min at RT. Slides were then incubated with anti-DIG-POD (1:400 in dilutant solution containing 1% sheep serum, 0.05% Tween-20, 1% BSA in PBS) for 60 min at RT. Finally, TSA™ Plus Fluorescein substrate (Perkin Elmer) substrate was applied to the sections and incubated 2 × 5 min at RT. PBS buffer washes were used to stop the reaction. The slides were covered directly with SlowFade Gold antifade reagent (Life technologies–Thermo Fisher Scientific) and examined under a confocal microscope (Leica TCS-SL), with suitable filter set.

To perform the densitometric analysis of FISH staining, six slices for each experimental group were considered. Signal intensity of miRNAs and U6 expressions were measured using ImageJ (https://imagej.nih.gov/ij/) to calculate the product of area and mean gray value (integrated density) as percentage of modulation (miRNA percentage value versus U6).

### Morphological Analyses

Twelve rats (*n* = 3 for each group) were deeply anesthetized with sodium tiopenthal (via i.p.) and transcardially perfused with physiological saline followed by 4% PFA in PBS. Brains were removed, postfixed in 4% PFA for 48 h, and then transferred in PBS. Vibratome was used to cut brains in serial sections (50 μm thick), which were collected in PBS and mounted on slides. FluoroJade staining was performed as previously described [29]. Briefly, slides were sequentially immersed in 100% ethanol and then in 70% ethanol. After rinsing in distilled water, slides were incubated in 0.06% KMnO_4_ solution, rinsed in distilled water and transferred to a 0.001% solution of FluoroJade dissolved in 0.1% acetic acid. Slides were then rinsed in distilled water, dried, immersed in xylene and coverslipped with Entellan. FluoroJade-positive cells were observed by Axioskop fluorescence microscope (Carl Zeiss, Germany) equipped with a filter suitable for visualizing fluorescein isothiocyanate; cells with evident neuronal morphology were counted in at least six sections for each animal taken along rostro-caudal extension of CA1 hippocampal field. Eighty-five fields/animal (270 × 250 μm each) were considered.

### Statistical Analysis

Data were expressed as mean ± SEM. For multiple variable comparison, results were analyzed using appropriate ANOVA test (one-way or two-way) followed by Tukey’s post hoc test; for single comparison, Student’s *t* test and *χ*^2^ test, unpaired *t* test, or Wilcoxon signed-rank test, after normal distribution testing (Shapiro-Wilk test) were appropriately used. Significance threshold was established for *p* = 0.05.

## Results

### Electrophysiological Recordings

#### Population Spike Analysis

In order to assess differences in network excitability in hippocampus of non-epileptic and epileptic rats with and without α-T post-ictal treatment, stimulations of Schaffer collaterals in *stratum radiatum* of brain slices and recordings from CA1 pyramidal cell layer were performed.

**Fig. 1:**
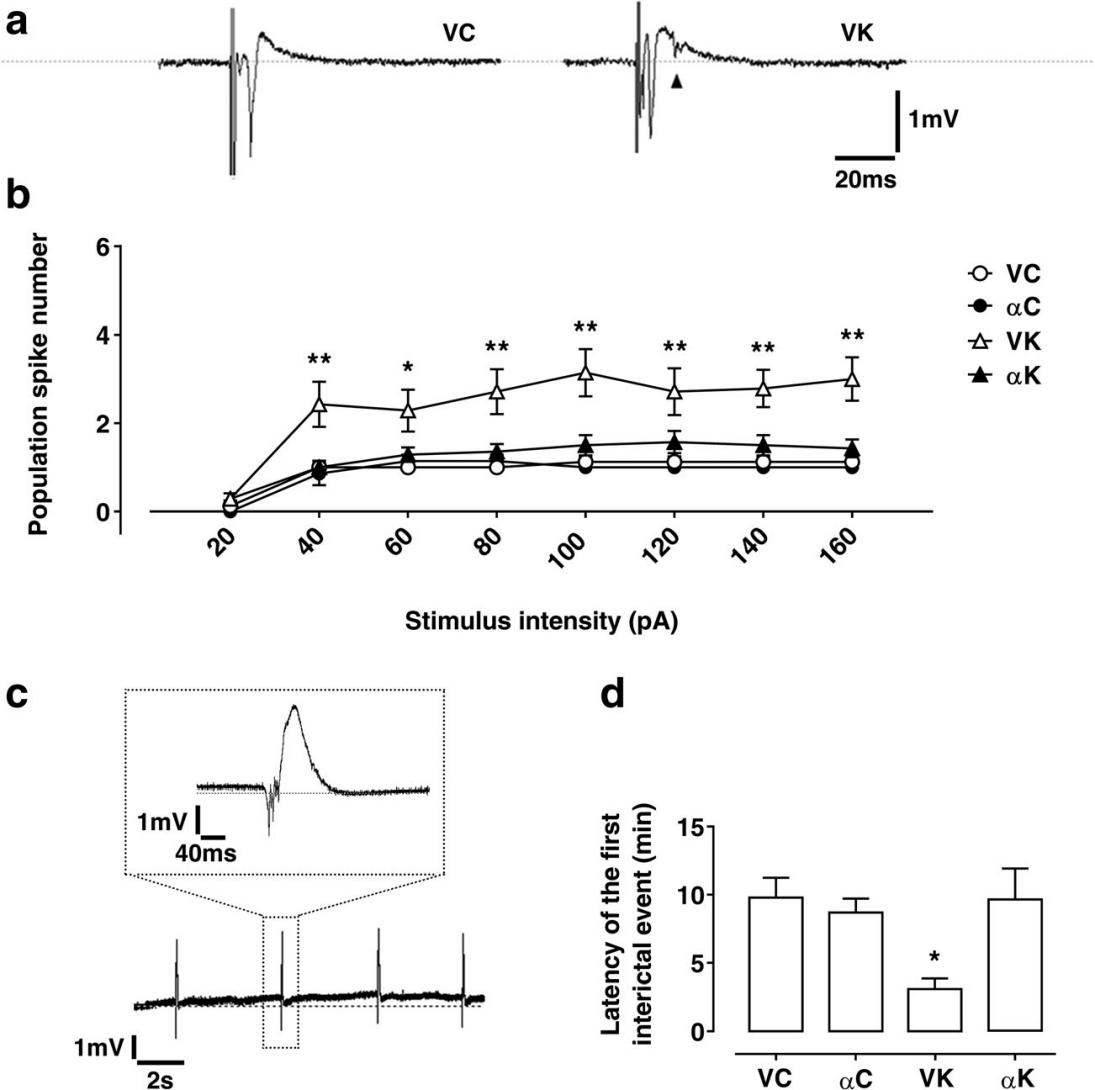
Effect of α-tocopherol treatment on slice excitability in control and kainate-induced rats. **a** Field excitatory postsynaptic potential (fEPSP) recorded in CA1 pyramidal layer in treated kainate-exposed (αK) and untreated kainate-exposed (VK) rats. The arrowhead indicates multiple population spike recorded in VK rat slice. **b** fEPSP population spike number recorded in the experimental groups: αK, treated kainate-exposed; VK, untreated kainate-exposed; αC, treated non-epileptic; VC, untreated non-epileptic, in response to Schaffer’s collaterals stimulation (increasing intensities from 20 to 160 pA). Statistical analyses performed by two-way ANOVA repeated measure, Tukey’s post hoc: *p < 0.05; **p < 0.01. **c** Bath application of 50 μM BMI together with 50 μM 4-AP leads to the appearance of spontaneous interictal events: on the top, the enlarged view of a single event is reported. **d** Latency of the first interictal event recorded in the experimental groups. Statistical analyses performed by one-way ANOVA, Tukey’s post hoc: *p < 0.05. All data are expressed as mean ± SEM

In non-epileptic rats (αC and VC), extracellular stimulation, during baseline recording, typically evoked a fEPSP characterized by a single population spike (Fig. 1a); a second population spike was rarely found in these groups of rats in response to Schaffer collaterals stimulation (Fig. 1b).

Conversely, slices from kainate-treated rats showed a fEPSP, which gave rise to one to three/four population spikes (Fig. 1a; VK mean value 2.71 ± 0.24); notably, only slices from VK rats exhibited up to four population spikes, while a great number of αK slices evoked only one population spike, thus similarly to non-epileptic rats (αK mean value 1.68 ± 0.17). Moreover, to better investigate neuron excitability, the number of population spikes was analyzed performing an input/output curve at increasing stimulus magnitude; results showed that VK group displayed a significantly higher number of population spikes compared to αK, starting from 40 pA stimulus intensity (Fig. 1b), indicating the occurrence of a greater network excitability in absence of α-T post-ictal treatment.

#### Induced Epileptiform-Like Network Activity

Spontaneous appearance of epileptiform bursting events was exclusively found in about 40% of recorded slices (*n* = 15) of VK groups (*χ*^2^ test, *p* < 0.01), supporting the higher hyperexcitability of the hippocampal network in this group of rats.

When BMI and 4-AP were co-applied in perfusion bath, epileptic-like activity was induced in slices. The adopted blocker concentrations were able to mainly evoke interictal events, characterized by a duration of less than 400 ms, with a positive peak and a negative one, clearly distinguishable from baseline activity (Fig. 1c); ictal event, defined as constituted by at least three interictal events in rapid succession, were rarely observed. As index of hyperexcitability, we evaluated the latency for the onset of the first interictal event. Epileptic rats treated with α-T showed a latency for inducing the first interictal event very similar to that of non-epileptic rats with and without treatment, and significantly different from that detected in non-treated epileptic rats (Fig. 1d), indicating a less marked hippocampal excitability under α-T treatment following SE.

#### GABA_A_R and AMPA Current Evaluation in *Xenopus* Oocytes

We tested the effect of α-T treatment on electrophysiological properties of GABA_A_ and AMPA receptors taking advantage of the technique of membrane microtransplantation in *Xenopus* oocytes.

First, we obtained a good expression of GABA_A_ and AMPA receptors using tissues from both treated and untreated rats (mean I_GABA_—81.2 ± 26.5 nA VK, *n* = 14 vs. 101.8 ± 31.4 nA αK, *n* = 13, *p* = 0.369; mean I_AMPA_—75 ± 33.5 nA VK, *n* = 10 vs. 118.5 ± 43 nA αK, *n* = 14). This small variability in the current amplitude is due to the difference of expression between cells and donors (frogs) as previously reported [44, 45]. Furthermore, we focused on the evaluation of the electrophysiological properties of the two receptors. As for GABA_A_R, we found that a significant current rundown was present in both αK and VK hippocampal tissues (43 ± 7.5%, VK, *n* = 10 vs. 54.8 ± 6.9%, αK, *n* = 12, *p* = 0.259), suggesting that α-T does not act on the degree of current desensitization as previously reported for BDNF or fracktaline [37, 46].

Additionally, no differences of E_GABA_ were observed between the two groups (VK = − 24.7 ± 1.2 mV; αK = − 22.6 ± 1.4 mV; *p* > 0.05 (*n* = 10)), thus indicating that a modification of chloride homeostasis is not likely to be involved in the effect evoked by the administration of α-T.

Furthermore, we analyzed I_AMPA_ normalized to I_GABA_ amplitude [47], but this parameter was similar in the two groups (AMPA/GABA—VK = 304 ± 82%; αK = 239 ± 47%, *p* > 0.05; *n* = 10).

To further characterize AMPA responses, we tested IEM1460, a selective GluR2-lacking AMPA receptor blocker, since GluR2 function is crucial in development and various pathologies [40]. Our results showed that the percentage of IEM1460 block was similar in the two groups (VK = 60.3 ± 16%; αK = 53 ± 14; *p* > 0.05, *n* = 8) indicating no difference in the expression of GluR2 between two groups of rats.

## Biochemical Results

### Oxidative Stress Evaluation

**Fig. 2:**
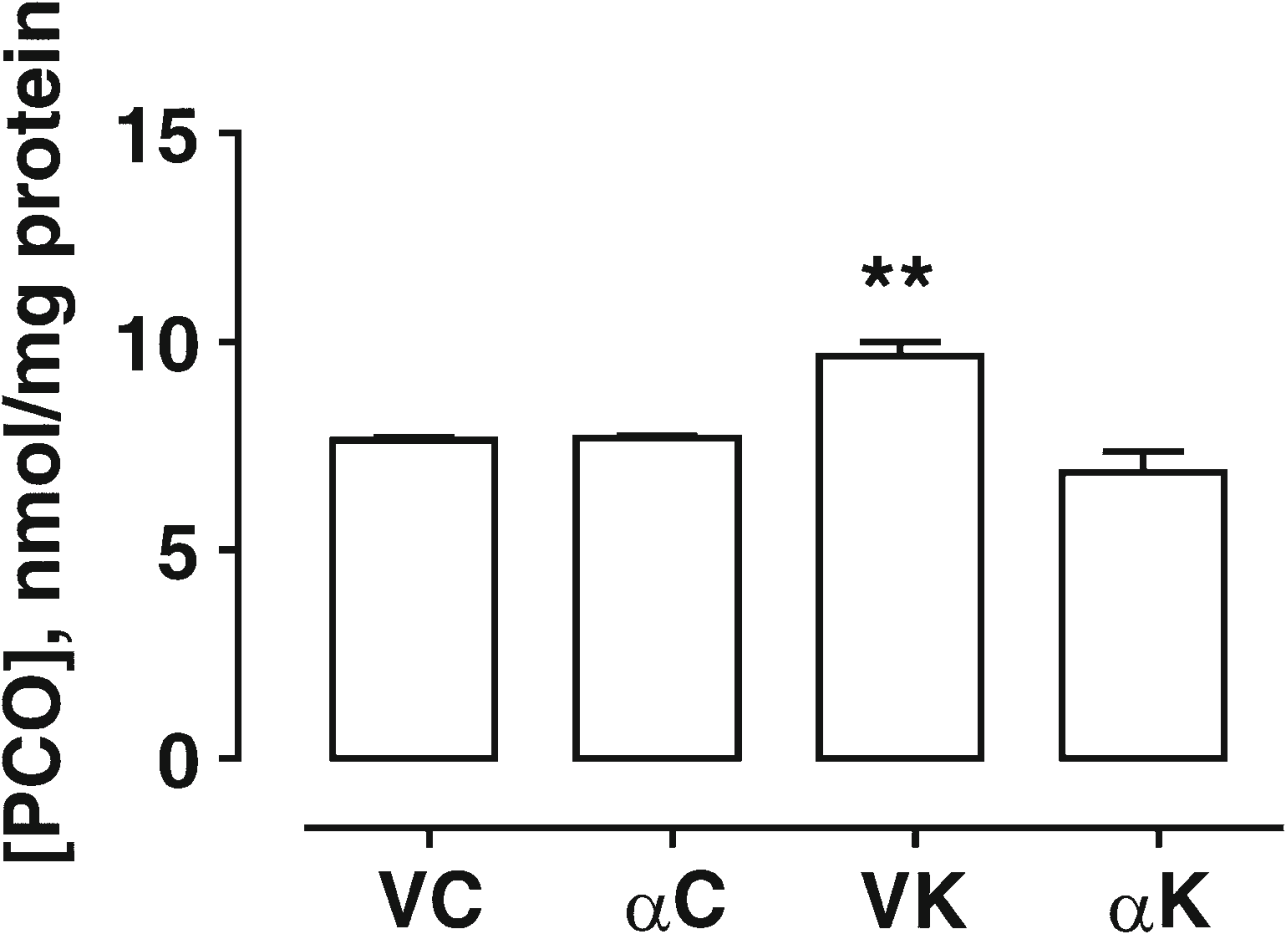
Effect of α-tocopherol treatment on protein carbonyls (PCO) formation used as marker of oxidative stress. PCO content (nmol/mg protein) in the hippocampal homogenates obtained from rats of different experimental groups: αK, treated kainate-exposed; VK, untreated kainate-exposed; αC, treated non-epileptic; VC, untreated non-epileptic. Histograms represent PCO content of three independent measurements in each experimental group (means ± SEM). Statistical analyses performed by one-way ANOVA and Tukey’s post hoc test: **p < 0.01

The formation of PCO has been considered a marker of oxidative stress in hippocampi derived from the different experimental groups. In detail, the hippocampi of kainate-induced epileptic rats (VK group) showed a significantly larger amount of PCO as compared to α-T-treated epileptic group (Fig. 2), which in turn resulted similar to that detected in non epileptic rats. Otherwise, no difference was found in PCO content when α-T was administered under control conditions. These findings indicate a reduction in oxidative stress levels under α-T treatment conditions 15 days following SE.

### Neuroinflammation Assessment

**Fig. 3:**
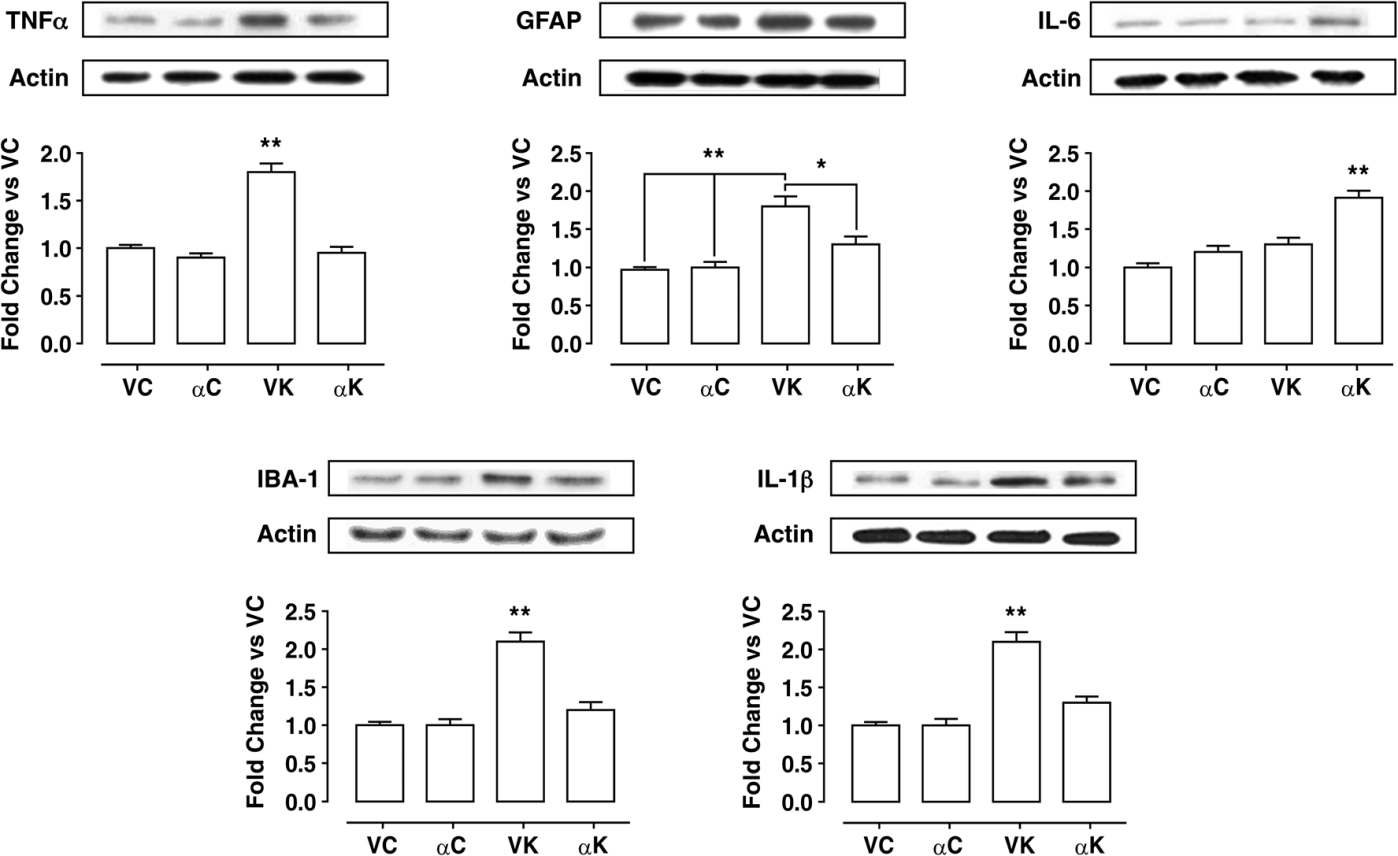
Effect of α-tocopherol treatment on neuroinflammatory markers in control and kainate-induced epileptic rats. Western blot analysis of the expression levels of neuroinflammatory markers in hippocampal homogenates obtained from rats of the experimental groups: αK, treated kainate-exposed; VK, untreated kainate-exposed; αC, treated non-epileptic; VC, untreated non-epileptic. Per each marker protein: representative immunoblots are displayed and anti-actin blots are shown as loading control. Note that immunoblots are shown in the same sequence as bars in the corresponding histograms. Histograms represent densitometric analyses of blots from three independent experiments (means ± SEM). Statistical analyses performed by one-way ANOVA and Tukey’s post hoc test: *p < 0.05; **p < 0.01

Fifteen days after SE, the expression levels of GFAP and IBA-1 (used as a marker of reactive astrogliosis and of microglial activation, respectively) were both significantly increased in VK animals when compared to VC rats (Fig. 3); consistently, the expression of pro-inflammatory cytokines IL-1β and TNF-α was also up-regulated (Fig. 3), whereas IL-6 protein levels appeared to not change. In αK rats, all the neuroinflammatory markers were expressed to a significantly lower extent than in VK (Fig. 3), except IL-6 which significantly increased.

**Fig. 4:**
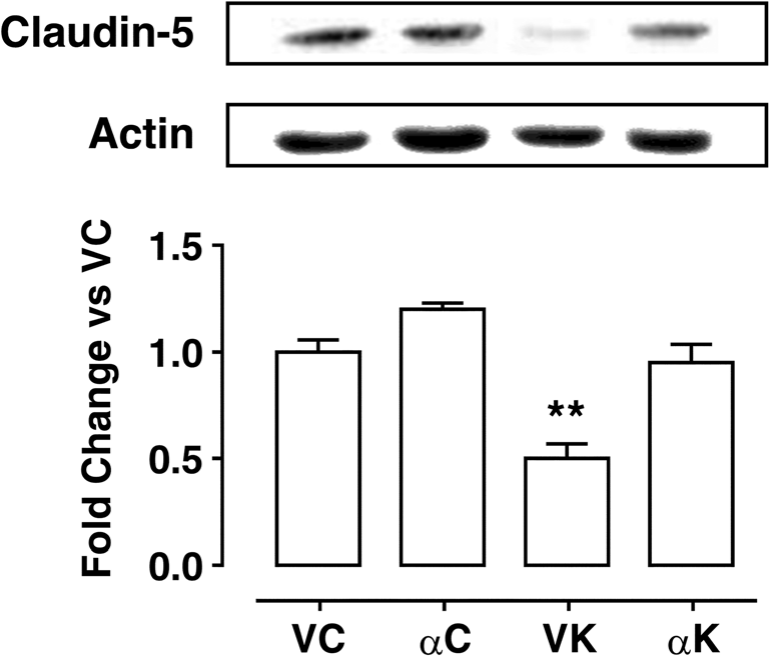
Effect of α-tocopherol treatment on blood–brain barrier in control and kainate-induced epileptic rats. Western blot analysis of the expression levels of claudin-5, used as marker of blood–brain barrier integrity, in hippocampal homogenates obtained from rats of the experimental groups: αK, treated kainate-exposed; VK, untreated kainate-exposed; αC, treated non-epileptic; VC, untreated non-epileptic. Per each experimental group: representative immunoblots are displayed and anti-actin blots are shown as loading control. Note that immunoblots are shown in the same sequence as bars in the corresponding histograms. Histograms represent densitometric analyses of blots from three independent experiments (means ± SEM). Statistical analyses performed by one-way ANOVA and Tukey’s post hoc test: **p < 0.01

As a consequence of neuroinflammation, disruption of the blood–brain barrier can occur, losing its capability to protect brain environment. Claudin is a molecule forming tight junctions, which limit the paracellular permeability. Thus, we evaluated the expression levels of this protein in hippocampi in order to gain insight regarding the integrity of the blood–brain barrier 15 days after SE. Densitometric analysis of immunoblots revealed a drastic decrease in claudin-5 protein levels in kainate-exposed rats, which was recovered by α-T treatment (Fig. 4).

## MicroRNA Expression Modulation

Aberrantly expressed microRNAs that have already been detected in hippocampus of epileptic patients (mTLE) and TLE mice models (pilocarpine-induced SE) [48], included miR-124, miR-126, and miR-146a. In particular, recent studies draw attention on the role of miR-146a in the regulation of astroglia-mediated inflammatory response [49], revealing its overexpression in glioneuronal lesions from patients with medically intractable epilepsy. In addition, miR-146a has been found to modulate IL-6 expression by targeting IRAK-1 and TRAF-6, key regulator proteins of TLR-4 signaling activation [43].

**Fig. 5:**
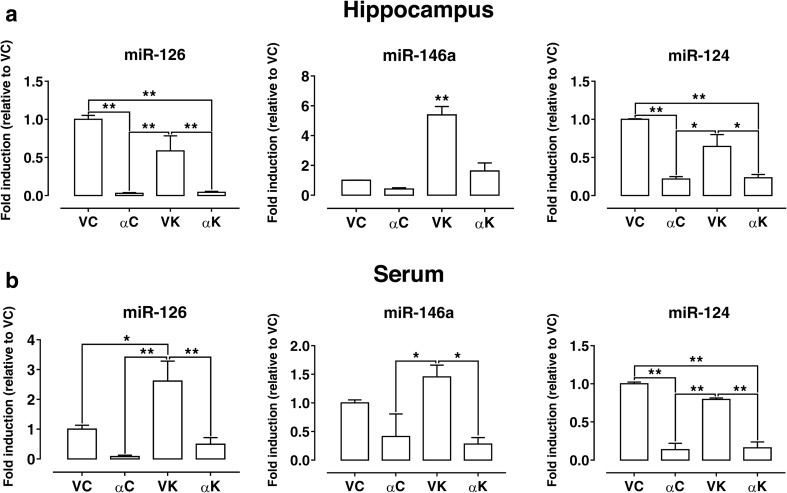
Effect of α-tocopherol treatment on miR expression. Quantification of miR-126, miR-146a, and miR-124 expression in hippocampal homogenates (**a**), and quantification of serum miR-126, miR-146a, and miR-124 expression (**b**) obtained from rats of the different experimental groups: αK, treated kainate-exposed; VK, untreated kainate-exposed; αC, treated non-epileptic; VC, untreated non-epileptic. Statistical analyses performed by one-way ANOVA and Tukey’s post hoc test: *p < 0.05; **p < 0.01. All data have been normalized on VC miR expression level and expressed as mean ± SEM

Thus, to test for the differential expression of the selected three miRNAs, such as miR-124, miR-126, and miR-146a, among our experimental groups, we isolated microRNAs from hippocampal homogenates. We detected a significant up-regulation of miR-146a in hippocampi of non-treated epileptic rats (VK) (Fig. 5a) as compared to non-epileptic ones (VC), but this SE-induced effect was completely prevented by α-T treatment (αK) (Fig. 5a). On the contrary, a trend of decrease in miR-126 and miR-124 expression levels was observed in epileptic hippocampi in comparison to VC; after α-T treatment, the differences became statistically significant. Interestingly, this effect was induced by α-T also in absence of epileptic insult (αC).

Moreover, we measured circulating microRNAs in the serum of the same rats belonging to the different experimental groups. We uncovered changes for miR-146a and miR-124 that fit with those observed in the related hippocampi (Fig. 5b); on the contrary, miR-126 serum levels were strongly up-regulated following SE (VK), a phenomenon promptly prevented by α-T treatment.

**Fig. 6:**
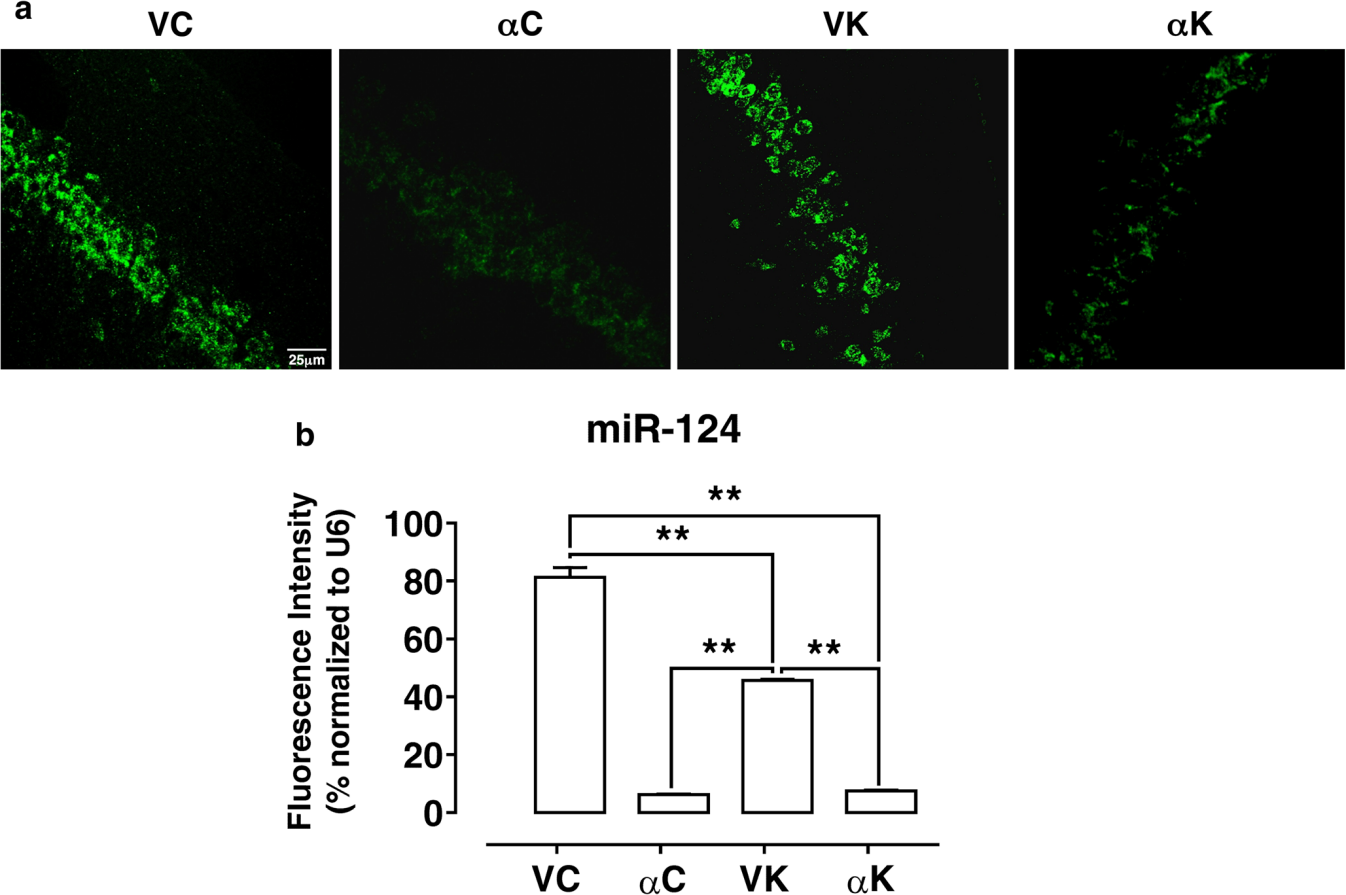
Effect of α-tocopherol treatment on miR-124 expression. Representative confocal FISH images of fluorescent in situ hybridization of miR-124 in CA1 pyramidal cells (scale bar 25 μm) (**a**). Quantification of FISH intensity of miR-124 normalized to U6 snRNA in CA1 pyramidal cells (**b**). αK, treated kainate-exposed; VK, untreated kainate-exposed; αC, treated non-epileptic; VC, untreated non-epileptic. All data are expressed as mean ± SEM. Statistical analyses performed by one-way ANOVA and Tukey’s post hoc test: **p < 0.01

As far as miR-124 expression is concerned, both in hippocampus and in serum, its expression was significantly down-regulated by α-T. To further investigate its low hippocampus expression, the fluorescence miR-124 in situ hybridization was performed in pyramidal neurons of CA1 hippocampal fields. Even by using this technique, we confirmed its down-regulation. MiR-124 signal was bright in pyramidal cells of CA1 hippocampal field and restricted to cytoplasm (Fig. 6). The brightness of the FISH signal was quenched in neuron cytoplasm of α-T treated epileptic rats and was very similar to that of the corresponding α-T non-epileptic ones (Fig. 6).

All together, these results suggest an influence of α-T on microRNA expression. In addition, circulating microRNAs indicate that miR-126 and miR-146a may be biomarkers for the epileptic disease and useful in the evaluation of the effectiveness of treatment.

## FluoroJade Analysis

**Fig. 7:**
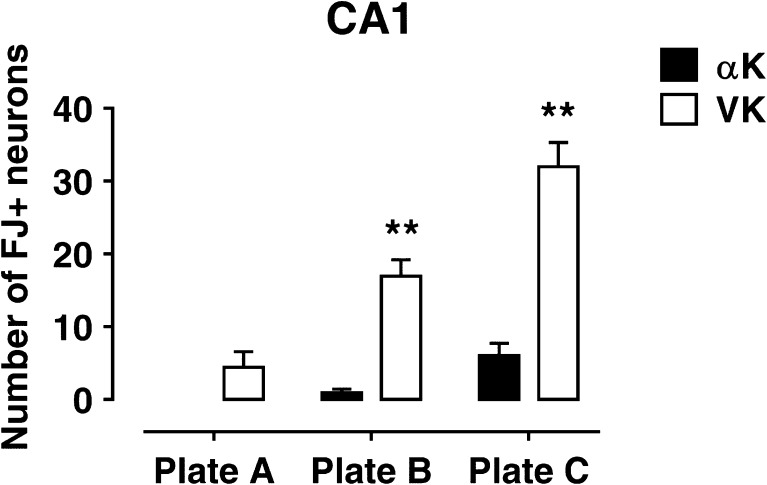
Effect of α-tocopherol treatment on neuronal degeneration. Histogram reports the quantification of FluoroJade-positive cells in CA1 hippocampal field in the different experimental groups. αK, treated kainate-exposed; VK, untreated kainate-exposed. Plate A, − 3.3 mm; plate B, − 4.3 mm; and plate C, − 5.8 mm from bregma. Statistical analyses performed by Student’s *t* test: **p < 0.01

Degenerating neurons were detected by FluoroJade staining. Fifteen days following SE, epileptic hippocampi showed a number of FluoroJade-positive cells, while no degenerating neuron was found in saline-injected rats (αC; VC). VK rats exhibited an overall greater number of degenerating neurons across rostro-caudal extension of CA1 field (Fig. 7) than αK hippocampi, which was highly significant at more caudal brain levels (Fig. 7). This finding indicates a reduction in neuronal degeneration due to α-T post-seizure treatment, thus confirming and extending our previous finding [29].

## Discussion

TLE, the most common form of partial epilepsy in humans, is often preceded by a latent period, free from seizure, during which maladaptive network reorganization occurs, thus inducing the appearance of spontaneous recurrent seizures that characterize the chronic stage [50]. Kainate status epilepticus mimics the TLE disease [51], thus representing a validated animal model to study the onset and development of chronic epilepsy. The CA1 region of hippocampus following kainate-induced SE is hyperexcitable, establishing a permissive factor for the genesis and propagation of epileptic seizures. The present study demonstrates that α-T treatment, started a few hours after the overt SE, is able to strongly reduce CA1 hippocampal neuron network excitability, decreasing the number of population spikes evoked in slices by field recordings and reducing the susceptibility to generate spontaneous and drug-triggered epileptiform bursting events as observed 15 days after initial insult; these electrophysiological parameters are considered good markers in the evaluation of neuronal network excitability during epileptogenesis [33, 52]. These findings would support the use of vitamin E in improving seizure control in patients with epilepsy [20], when added to the antiepileptic drugs (AEDs). However, the mechanisms underlying the antiepileptogenic effect of this natural compound have not yet been fully investigated. Since a number of studies have shown an increase in oxidative stress in epilepsy [53, 54], vitamin E effectiveness in mitigating epilepsy severity in human patients and in animal models has been ascribed to its well-known antioxidant property [19, 20]. By the way, our findings regarding PCO determinations indicate the significant reduction of oxidative stress level in hippocampus of α-T-treated epileptic rats. Nevertheless, a series of studies revealed that the forms of vitamin E, mainly α-T, are able to function as signaling and gene regulation molecules independently from their antioxidant action [55] and in the last few years, several laboratories have described additional cellular and molecular properties for this vitamin, such as, reduction of proliferation rate [56, 57], the enhancement of immune functions, inflammatory pathway regulation, and neuroprotection [29, 58–61].

### α-Tocopherol Reduces Hippocampal Neuroinflammation and Neurodegeneration

Neuronal cell loss, dendritic synapse elimination and synaptic reorganization, imbalance in excitatory and inhibitory drive, gliosis, and inflammatory pathway activation may contribute to alter network excitability and synchronization during the seizure-free latent period, accounting for epileptogenesis. In this view, our previous study [29] has shown that 4 days of α-T treatment after kainate-induced SE is able to (1) quench neurodegenerative and neuroinflammatory processes triggered by SE; (2) counteract glutamine synthase decline, recovering the glutamate–glutamine–GABA cycle pathway, which has been found to be disrupted in the hippocampus of patients affected by temporal lobe epilepsy (TLE) [62]; (3) induce an increase in dendritic spine number and in synaptophysin immunoreactivity, suggesting a role of α-T in synaptogenesis promotion and/or synapse protection.

The present findings confirm and extend our previous ones, showing that α-T treatment for 15 days following SE induction significantly reduce astrogliosis and microglial activation, lowering the hippocampal expression of glial and microglial markers and of pro-inflammatory cytokines IL-1β and TNF-α; on the other hand, IL-6 protein levels are up-regulated by α-T, whereas non-treated epileptic rats do not show any difference with respect to both groups of non-epileptic animals. This latter finding might be consistent with the time-course of IL-6 induction, which has been found to reach maximal levels within 24 h after SE and then decrease after 3 days [63, 64]. IL-6 is a multifunctional cytokine with pro- but also anti-inflammatory activities [65], and it has been reported to contribute to neuroprotection after status epilepticus [66]. In addition, the absence of IL-6 appears to be responsible for a higher seizure susceptibility to some chemoconvulsant agents [67]. Mechanisms underlying IL-6 neuroprotective effect have not been fully elucidated, but it has been demonstrated to down-regulate the synthesis of pro-inflammatory cytokines IL-1β and TNF-α [68, 69], thus limiting neuroinflammation contribution to epileptogenesis. In keeping with these observations, here we found a low protein expression level of IL-1β and TNF-α in epileptic rats treated with α-T as compared to the corresponding non-treated ones that could in part come from their down-regulation induced by IL-6. Interestingly, the small regulatory RNA molecule miR-146a, an inflammation-associated microRNA up-regulated during epileptogenesis and in the chronic epileptic phase in rat hippocampus [70], has been recognized as an endogenous regulator of cytokine signaling [71]. In this view, He et al. [72] demonstrated that miR-146a inhibitor increased, while miR-146a mimics decreased, the expression of IL-6 in LPS-stimulated macrophages. In our experimental model, we found that miR-146a expression was significantly enhanced in VK rats in comparison to normal rats, and α-T treatment was able to restrain miR-146a up-regulation in epileptic rats 15 days after SE induction. Considering that α-T was previously disclosed to down-regulate miRNA levels [24], we hypothesize that it may be able to reduce miR-146a expression in rat hippocampus during the latent period following SE induction and, as a consequence, to increase IL-6 release. Moreover, it is worth mentioning that other mechanisms could be involved in the neuroprotective effects of IL-6; indeed, it was found to facilitate the concentration- and time-dependent up-regulation of adenosine A1 receptor mRNA and signaling [73] and adenosine has been shown to exert a powerful anticonvulsant effect [74]. In addition, in vitro studies showed that IL-6 is able to protect against glutamate- and NMDA-induced excitotoxicity [75, 76].

Our present findings also corroborate the protection exerted by α-T toward SE-induced neurodegeneration. Degenerating neurons were scattered throughout the rostro-caudal CA1 extension, but they were mainly gathered at more caudal hippocampal levels, in which we detected a significantly lower number of degenerating neurons in α-T-treated epileptic rats in comparison to the corresponding non-treated ones. The rostro-caudal gradient in vulnerability of CA1 to epileptic injury could be consistent with the increasing degree of neuronal excitability from dorsal to ventral hippocampus described by several authors [77–81], and it is noteworthy that post-ictal α-T administration is able to rescue CA1 neurons, showing a corresponding region-specific pattern along the rostro-caudal axis of hippocampus.

### α-Tocopherol Promotes Blood–Brain Barrier (BBB) Recovery

The BBB is a highly selective semipermeable membrane barrier required to separate the circulating blood from brain extracellular fluid in the central nervous system (CNS); this ability is attributed to endothelial cells, which have continuous tight junctions, lack fenestrations, and are characterized by low pinocytotic activity. A compromised functioning of brain endothelial cells results in serious consequences for BBB integrity [82] and therefore for brain homeostasis. BBB breakdown can be a direct consequence of seizure activity [83] due to pro-inflammatory cytokine surge [27], inducing shifts in vascular physiology, which is accompanied by entry into the CNS of blood-borne entities ranging from small molecules to whole cells [84, 85]. Tight junctions contain a complex of transmembrane proteins, including claudin, the alteration of which in the expression levels is indicative of a BBB damage.

We believe that this study shows, for the first time in this experimental model, the ability of α-T to promote BBB recovery, probably by reducing neuroinflammation levels after SE. This is a remarkable effect considering the crucial role played by BBB in maintaining cerebral homeostasis and providing neuroprotection.

### α-Tocopherol Affects microRNA Expression in Hippocampus

Much evidence indicates that numerous microRNAs are dysregulated in epileptic brain of human and animal models [86]. In keeping with these studies, here we found that miR-146a was significantly up-regulated in hippocampus of non-treated epileptic rats 15 days after SE induction, but α-T treatment was able to prevent its overexpression. On the contrary, miR-126 and miR-124 were significantly down-regulated in α-T-treated epileptic hippocampi. It is remarkable that all the considered miRs showed an alteration of their expression under α-T treatment also in absence of the epileptic insult, thus indicating a possible direct effect of the vitamin on miR expression. Consistently, a redox-independent down-regulation of microRNA expression by α-T has been previously reported [24].

The miR-146a appears to be a crucial mediator in the neuroinflammatory response and it has been demonstrated [70] that an up-regulation of miR-146a occurs in the latent period of epileptic disease both in rat model and in human TLE, thus suggesting a potential role played by these miRNAs in pathogenesis of the disease. The expression of miR-146a is induced by pro-inflammatory cytokines, mainly IL-1β [71, 87], and, in turn, it has been suggested to act in a “negative regulatory loop” [88] to fine-tune inflammatory response. In this context, we may hypothesize that α-T, since it decreases the expression levels of IL-1β and TNF-α pro-inflammatory cytokines, is able to prevent miR-146a up-regulation induced by SE, and that the reduced expression of miR-146a may increase, as discussed above, IL-6 release, which contributes to further down-regulate IL-1β and TNF-α protein levels.

miR-126 is highly expressed in endothelial cells, playing a critical role in angiogenesis and blood vessel integrity [89, 90]; moreover, miR-126 has been demonstrated to regulate the expression levels of endothelial adhesion molecules in inflammation [91]. Recent findings uncovered a crucial role for miRNAs, among which miR-126, in controlling the function of the barrier endothelium in the brain, which can be reduced by pro-inflammatory cytokines [27]. Here, we found that miR-126 expression level tends to decrease in hippocampus 15 days following SE; this variation could be related to neuroinflammation occurrence which impairs BBB integrity as shown by the dramatic loss of claudin-5 that we detected in epileptic non-treated rats. Curiously, under α-T treatment conditions, we observed a stronger decline of miR-126, but independently from the induction of epileptic insult. This finding suggests that α-T per se may be a powerful regulator of miR-126 expression in brain, thus affecting its downstream effectors, including those related to cell growth and survival pathways [92]. Indeed, miR-126 has been validated as a regulator of SOX2 [93], which in the neural context is an important factor in maintaining self-renewal and pluripotency of stem cells and neural progenitors [94]. In addition, it is involved in EGFL7 pathway, which regulates Notch signaling in neural stem cells altering their self-renewal and multipotency as well as their differentiation potential [95]. Taking into account that we performed miR-126 evaluation in the whole hippocampus, including dentate gyrus in which neurogenesis occurs in normal and pathological conditions such as epilepsy, and that previously we found that α-T per se is able to modulate dentate gyrus neurogenesis [96–98], our results could be affected by neurogenetic events altered by epilepsy and treatment.

miR-124 is abundantly and specifically expressed in the whole brain, and growing evidence is underpinning the link between miR-124 dysregulation and central nervous system (CNS) disorders [99]. Consistently, miR-124 has been demonstrated to be useful as a diagnostic and prognostic indicator of CNS pathologies, such as brain tumor and stroke [99]. Recently, miR-124 has been described to be involved also in epilepsy in which it seems to play dual and opposing roles in epileptogenesis; indeed, if on one hand, it has been shown to attenuate epileptogenesis by preventing neuron-restrictive silencer factor (NRSF) up-regulation involved in the development of epilepsy, on the other hand, miR-124 exacerbates inflammation promoting epilepsy [26]. In our experimental conditions, we found that hippocampal miR-124 expression was not significantly modified 15 days after kainic acid-induced SE, even though it showed a trend toward down-regulation. We further performed miR-124 FISH analysis, and we found it to be expressed in cytoplasm of hippocampal neurons, including pyramidal neurons of CA1 field. Both RT-PCR quantitative analysis and FISH staining showed that under α-T treatment conditions, miR-124 expression was significantly down-regulated 15 days following SE, but it was similar to that of the corresponding non-epileptic rats, thus suggesting a direct action of α-T per se also on miR-124 expression. However, considering the effect of α-T on neuron network excitability, these findings could support a pro-epileptogenic role played by miR-124 in epilepsy.

### α-Tocopherol Affects MicroRNA Expression in Serum

The expression levels of microRNAs in blood have been recognized to be clinically relevant as diagnostic/prognostic biomarkers for a number of human diseases [100]. In keeping with this evidence, we found that the modulation of serum miR-124 and miR-146a expressions in the different experimental rat groups overlaps that observed in hippocampi from the same animals, closely reproducing alterations induced by SE and α-T treatment. In particular, miR-124 deserves attention since it is specifically expressed in brain and it has been shown to be useful as a diagnostic and prognostic tool in some brain disorders. On the other hand, miR-126 evaluation in serum from epileptic rats without α-T treatment unveiled an opposite trend in comparison to that found in the corresponding hippocampi, and it showed a significant up-regulation with respect to miR-126 serum levels detected in all the other experimental groups. Notably, this finding also demonstrates the reproducibility of the α-T effect on miR-126 expression.

Thus, we believe that our findings allow us to propose that the considered circulating microRNAs could serve as biomarkers to follow up the effectiveness of the therapy for the treatment of epilepsy.

### α-Tocopherol Does Not Influence GABA_A_R and AMPA Current

Imbalance in excitatory and inhibitory drive represents one of the pathological mechanisms underlying the progression of seizures, so that GABA, the most important inhibitory neurotransmitter in the CNS, and its receptors are considered the main target of current and future antiepileptic drugs.

The occurrence of recurrent seizures is closely related to a reduced efficacy of GABAergic inhibition [101]. Indeed, epileptic hippocampus has been found to show GABA_A_ receptors which become less responsive to repeated activation than those from normal tissue both in humans and in animal model of epilepsy [102–106]. This GABA_A_ receptor desensitization (known as GABA_A_R rundown) may be a crucial mechanism in disrupting inhibitory function, contributing to excitation–inhibition imbalance [25] and thus accountable for an increase in excitability and for the occurrence of spontaneous seizures. In this context, we found that α-T did not attenuate the SE-induced degree of GABA current desensitization, displaying both αK and VK hippocampal tissues a very similar marked rundown during repetitive applications of GABA. Although the molecular mechanisms underlying the increased GABA_A_-receptor rundown in the epileptic tissue are still unknown, it has been hypothesized that it could be ascribed to alterations in GABA_A_ receptor subunit composition [102] or to changes in GABA_A_R phosphorylation/dephosphorylation [25]. Thus, based on our results, we may suppose that α-T is unable to affect GABA_A_ergic receptor population structure nor their function regulation. In addition, α-T post-ictal administration does not seem to influence chloride homeostasis, suggesting that chloride transport expression and/or activity are not altered by the molecule administration. Finally, AMPA/GABA current ratio was not different in the two groups, as well as AMPA response and subunit composition, indicating no effects directly exerted by α-T also on AMPA expression and/or functioning.

## Conclusion

In the present work, we used a multidisciplinary approach for the evaluation of the antiepileptogenic effect of α-T isoform of vitamin E and to investigate neurobiological correlates underlying this action. α-T treatment during the seizure-free latent period after SE allows the reduction of maladaptive plasticity processes, including neuroinflammation and neurodegeneration, which promote the occurrence of spontaneous recurrent seizures, inducing chronic epilepsy. Our main findings unveil the actual and strong decrease in hippocampal network excitability following SE under α-T treatment conditions and the possible involvement of an α-T-mediated anti-inflammatory mechanism in reducing epileptogenesis.

Furthermore, epileptic rats did not show any adverse reaction linked to α-T treatment, such as significant weight loss, bleeding, diarrhea, liver structure damage, etc., suggesting the safety of α-T use as an antiepileptogenic agent. This is a very relevant aspect because the absence of side effects associated with any drug administration represents an essential positive feature; indeed, adverse side effects of current AEDs are a major impediment to optimal dosing for seizure control, mainly under drug-resistant epilepsy conditions. Hence, the search for medications that treat epilepsy with minimal or no side effects is gaining interest, and phytopharmaceuticals or alternative drugs or supplements are being considered as probable candidates.

Interesting results have been obtained with the analysis of miRNAs expression, but the α-T effect on microRNA modulation observed in hippocampus will have to be deeply investigated. Future experiments will be needed to discover the processes modulated by these miRNAs during our treatment conditions. The in vivo miRNAs manipulation could be of great support to further understand their direct involvement in the α-T antiepileptogenic mechanism of action observed in this study.

Finally, up-regulated circulating miR-126 and miR-146a during epileptogenesis may be not only valuable diagnostic/prognostic biomarkers but also useful biomarkers to evaluate the clinical efficacy of specific treatments.
